# Drivers of respiratory health care demand in Acre state, Brazilian Amazon: a cross-sectional study

**DOI:** 10.1186/s12889-022-14171-z

**Published:** 2022-09-24

**Authors:** Thiago Morello, Aldo Santos Lima, Rubicleis Gomes da Silva

**Affiliations:** 1 Centre of Engineering, Modelling, and Applied Social Sciences, Federal University of ABC, Alameda da Universidade, S/N, Bairro Anchieta, São Bernardo do Camp, SP 09606-045 Brazil; 2 Centre for Law and Applied Social Sciences, Federal University of Acre, Caixa-postal: 500, Distrito Industrial, Rio Branco, AC 69915-900 Brazil

**Keywords:** Respiratory illnesses, Health care seeking, Econometrics, Brazilian Amazon

## Abstract

**Background:**

The scarce knowledge about the drivers of demand for respiratory health care in the Brazilian Amazon, where the gap of human and physical health care resources is wide, is expanded with two surveys conducted in the west of the region, in Acre state. Potential drivers, informed by a review of twelve recent papers, were classified into seven categories capturing the individual, household, community and macroeconomic dimensions.

**Methods:**

Quantitative field surveys were conducted in 2017 and 2019 based on coupled conglomerate-quota randomization sampling. Adults responded about their own health or their children’s health. The probability of seeking physician care for the latest episode of respiratory illness or dry cough was analysed with multiple nonlinear regressions, having as covariates the potential predictors informed by the literature.

**Results:**

The propensity to seek health care and to purchase medication was larger for children. Influenza-like illness (Despite the exact diagnostic stated by respondents being “influenza”, a virus detection test (such as the PCR test) is not commonly applied, as informed by the Acre state public health service. In consistency, the term “influenza-like illness” is used.) was the most frequently diagnosed disease, followed by pneumonia, suggesting that a health care-seeking rate below 40% may perpetuate health impairment and local contagion. Illnesses’ severity, including the pain experienced, was the main predictor, revealing that subjective perception was more influential than objective individual and household characteristics.

**Conclusions:**

The results suggest that subjective underestimation of respiratory illnesses’ consequences for oneself and for local society could prevent health care from being sought. This is in line with some previous studies but departs from those emphasizing the role of objective factors. Social consequences, of, for instance, a macroeconomic nature, need to be highlighted based on studies detecting long-run relationships among health care demand, health and economic performance at the national level. Depending on the intensity of the trade-off between the costs imposed on the health system by increased demand and on the economy by the reduced productivity of the ill, policy could be adopted to change subjective perceptions of illnesses with nudges and educational and informational interventions.

**Supplementary Information:**

The online version contains supplementary material available at 10.1186/s12889-022-14171-z.

## Background

### Motivation

Respiratory illnesses are among the main sources of the global burden of disease according to [1], and the same is true in Brazil, where they are the third main cause of mortality [2]. A recent large international survey conducted by [3] with 5196 individuals from seven developed and seven developing countries, including Brazil, estimated that only 30% of patients with sore throat, a symptom associated with influenza-like illnesses and acute respiratory illnesses [4], seek a general practitioner. Similarly, high rates of refusal and delay in seeking care were found in national-level surveys [4–7]. With this, the impairment of one’s own health and labour productivity may be extended [8], and mortality and local contagion may be increased [5, 6].

Such negative social effects are more probable in less developed regions such as the Brazilian Amazon. There, the per capita number of families receiving poverty alleviation cash transfers is 1.5 times that of the whole country [9, 10],^1^ and large-scale exposure to pollution from agricultural fires is seasonal and comparable in its intensity to exposure to urban pollution in megacities such as London and Mexico City [11, 12]. Additionally, the prevalence of both tuberculosis (TB) and influenza-like illnesses is 1.3 times larger than the country’s average (of six cases per 10,000 inhabitants [10, 13];).^2^ At the same time, in the Amazon, the number of physicians per capita is thirtyfold below the world average, and 0.09 hospital beds per 1000 inhabitants are available, compared with 3.2 in the world [14, 15].

In addition to the supply-side gap, previous studies in Brazil also have detected demand-side barriers to health care, such as a low capacity to recognize, both in oneself and in children cared for, diseases, their severity and contagiousness [16, 17], a limited capacity to obtain and use health-relevant information, mainly by the low-educated [18, 19], household income incompatible with the transport costs required for visiting a health facility, which are more likely for rural residents [20], and reliance on self-medication [21].

Nevertheless, none of these studies is based on patient surveys uncovering factors enabling and disabling care seeking for respiratory illnesses. Such surveys are rare in Brazil and, as far as can be assessed, are not available for the Amazonian region. Nevertheless, to stimulate health care seeking, there is a need to detect patients’ characteristics predicting whether such a course of action would be pursued spontaneously or not. This is required for knowing which social groups should be prioritized by specific interventions [22], such as behavioural nudges [23] and educational campaigns [7], and how these must be designed. Seeking to fill such gaps, this study detects predictors of health care seeking among respiratory ill individuals in Acre state, western Amazon.

### Factors of demand for health care

Twelve recently published papers were consulted to identify potential factors influencing the demand for health care, as a basis for selecting covariates for quantitative analysis and for discussing and complementing the results. Summaries of all papers are provided in the Additional Information, and here, a short synthesis and taxonomy are presented.

The revised papers highlighted the relevance of multiple factors, including the characteristics of patients, social groups (households and communities), social contexts and macroeconomic features such as levels of development and economic growth (Table 1). These factors operate through multiple mechanisms at the individual (e.g., identification of illness from symptoms), household (e.g., women autonomy to seek care for children), community (e.g., support for seeking care) and macroeconomic levels (e.g., economic growth expanding national health systems). Therefore, the demand for health care is both a multidimensional and multilevel process that can hardly be apprehended with only one research method. This is clear from the larger diversity of factors in the literature compared with the datasets used in this paper (Table 1). Nevertheless, it also is clear that a significant fraction of factors is captured, with the uncaptured (macroeconomic) factors being left to be accounted for while discussing the results (Conclusions﻿). Additionally, the focus on demand-side variables is coherent with the consulted literature, especially with the finding by [5, 6] that delays in receiving TB treatment were mostly demand-driven.

**Table 1:** Classification of factors influencing demand for health care in previous studies and in this paper (demand enablers are indicated with “+” and disablers with “-”)

Papers	Class	Factors in the literature	Factors in this paper
DAT20, NAK20, SUK16, SEI18, NGH17, D20, TET17	Individual characteristics	.Education (+) [TET17] .Age (+) [SEI18, DAT20] (population ageing contributes to increased health expenditure at the macroeconomic level) (+) [NGH17] .Income (+) .Poverty (−)	.Education .Age .Income .Poverty .Gender
SUK16, ABD17, NGH17, CHE21, ZHA20	Health level and condition	.Chronic disease (+) .Interaction with ill individuals (+) .Illness stigmatization (−) .Anxiety (a positive predictor of TB stigmatization) [CHE21] (−) .Health level of the community and capacity to support health care seeking (+)	.Serious lung diseases (including chronic diseases) .Subjective pain level caused by respiratory illness or dry cough .Symptoms caused by respiratory illness .Duration of respiratory illness and dry cough
D20, DAT20, SEI18, SUK16	Health knowledge	.Ability to identify symptoms, their severity, linkage with illnesses and causes (+) .Limited knowledge about prevention and treatment (−) .Degree of “health literacy” or capacity to obtain and use information in order to ensure good health [SUK16] (+)	Proxied by education
D20, NAK20, CHE21, SUK16	Intrahousehold relationships, gender and communities	.Mothers’ agency to seek health care (−) and control of household budget (+); .Household size (−) .Female gender (−/+) .Support from relatives in seeking health care and overcoming stigmatization (+) .Social capital [SUK16] (+)	.Household size .Gender of respondent .Gender of child’s caregiver
D20, CHE21, NAK20, DAT20, DIA13	Access to care (including relationship with providers and treatment cost)	.Previous contacts (+) and good personal relations with health care provider (+), including good patient–physician communication (+) [CHE21] .Health facility distance (−) .Treatment cost (−), copayment or any out-of-pocket disbursement being required (−) .Opportunity cost of treatment (specifically transport cost) (−) .Waiting time (−)	.Facility distance or travel time .Waiting time .Has at least a moderate amount of time available to seek health care .Health insurance
D20, NAK20, TET17, DIA13, SEI18	Nonprofessional care (traditional medicine and alternative, self-prescribed and over-the-counter medication)	.Resort to self-made medication (including traditional plant- and herb-based remedies) (−) and self-medication with either traditional or ordinary drugs (−) [SEI18]; .Resort to traditional healers and “chemists” (medication vendors) (−) .Relative distance of formal and nonformal care options (e.g., “chemist”/drug vendors are inside the community and health facility is outside) (−)	Resort to nonprofessional health care (mostly traditional medication)
PIA17, NGH17	Macroeconomic factors	.GDP (predictor of public health care expenditure) [PIA17] (+) .Labour force (idem) [PIA 17] (+) .Technological progress (which fosters economic growth and thus health expenditure) [PIA 17] (+)	Not addressed

Citations are abbreviated as D20 ≡ [24], CHE21 ≡ [25], DAT20 ≡ [5], NAK20 ≡ [26], SUK16 ≡ [7], PIA17 ≡ [8], ABD17 ≡ [27], SEI18 ≡ [6], NGH17 ≡ [28], TET17 ≡ [29], ZHA20 ≡ [4], and DIA13 ≡ [22]. Detailed summary of papers in the Additional Information

### Region of study

Acre state, located in the western Brazilian Amazon, is among the least developed and health care-endowed states of Brazil, ranking 21^st^ of the countries’ 27 states in human development [30] and 23^rd^ in physicians per capita [10, 15]. Despite limited resources, respiratory illnesses are targeted by multiple actions, including annual vaccination campaigns [31] and the Family Health primary care programme, including home visits by community health agents (CHAs), who provide key advice on, for instance, home-based preventive measures, health care seeking and the administration of prescribed drugs.^3^ Medication for asthma and rhinitis also is subsidized [32].

The prevalence of respiratory illnesses in Acre state is summarized in Table 2, based on the most updated set of ambulatory visit records available at the time of the cough survey (2019). The four most frequent respiratory disease classes were, in decreasing order, (i) acute pharyngitis and tonsillitis, (ii) other acute infections of the upper airways, (iii) influenza-like illness or flu and (iv) pneumonia. Their joint frequency amounted to 78%. In complement, the TB incidence rate in Acre state was of 5.5 confirmed cases per 10 thousand inhabitants in the average across 2013 to 2019 [53, 54], a prevalence rate that was 13-fold smaller than that of pneumonia, one of the top four respiratory illnesses in Table 2; such comparison is nevertheless limited by TB frequency being based not only on ambulatory visits but also on visits to emergency rooms and hospitals. Regarding approximate aetiology, diseases caused by viral or bacterial pathogens (at least one of those) were most frequent both in Table 2 and based on respiratory mortality data for Acre state from 2013 to 2018 [55], with 56% of ambulatory visits and 47% of deaths (approximately 90% of the latter owed to pneumonia and approximately 9% to TB). Mixed (asthma), environmental and behavioural (smoking) sources accounted for 10% of morbidity and 42% of mortality, and this last figure was nearly entirely due to chronic obstructive diseases.

**Table 2:** Classification of respiratory disease episodes from Acre state ambulatory visit records, 2013–2018

Disease class ^**a**^	ICD-10 ^**b**^	Approximate aetiology ^**a**^	Aetiology source ^**a**^	Freq. ^**c**^
Asthma	J45-J46	Viral, bacterial, allergen-driven, pollution-exposure-driven but also genetic	[33–35]	4,08%
Acute bronchitis and bronchiolitis	J20-J21	Mainly viral, but bronchitis also may be bacterial	[36–39]	4,95%
Bronchitis, emphysema and other chronic obstructive lung diseases	J40-J44	Smoking and air pollution	[40–42]	6,12%
Chronic diseases of tonsils and adenoids	J35	Bacterial	[43]	0,01%
Acute pharyngitis and tonsillits	J02-J03	Bacterial or viral	[44, 45]	29,40%
Influenza or flu	J09-J11	Viral	[46]	14,38%
Acute laryngitis and tracheitis	J04	Bacterial or viral	[47–49]	0,26%
Other diseases of the respiratory system	J22, J66-J99	Indeterminate, as multiple diseases are included	Does not apply	6,69%
Other diseases of the nose and the paranasal sinuses	J30-J31, J33-J34	Idem	Does not apply	0,19%
Other diseases of the upper respiratory tract	J36-J39	Idem	Does not apply	0,00%
Other acute infections of the upper airways	J00-J01, J05-J06	Idem	Does not apply	26,77%
Pneumonia	J12-J18	Bacterial or viral	[50]	7,14%
Chronic sinusitis	J32	Bacterial	[51, 52]	0,001%
**Total**				100,00%

^a^Classification in the first column was made by Acre state health professionals and in the second column by the authors relying strictly on references reported in the third column (patient, clinical and laboratory information could not be accounted for in the aetiological classification for being not available)

^b^The respiratory disease classes in the table are those adopted in the Brazilian National Health System, which correspond to aggregations of ICD-10 categories

^c^Based on a total of 477,609 ambulatory visit episodes, approximately 79,000 episodes per year

## Method

### Respiratory illness survey

A structured quantitative survey was conducted in Rio Branco (~ 400 k inhabitants), the most populous town of Acre state, from March to July 2017, following a pilot wave in October–November 2016 with 146 participants. The goal was to elicit the willingness to pay (WTP) to avoid the recurrence of the most recent respiratory illness episode. A hypothetical vaccine was offered as a product capable of yielding full prevention. Since WTP estimates are not directly related to the goal of this paper,^4^ only the survey’s first module, on the description of the illness episode, and the third module, on the socioeconomic and health attributes, are detailed here.

The sample was selected based on a mix of conglomerate-quota randomization with the help of local CHAs (details are found in [56], SI, Section 4). In the first stage, ten neighbourhoods of Rio Branco’s urban perimeter were selected, initially randomly, and, afterwards, depending upon whether CHA support proved insufficient,^5^ new neighbourhoods were introduced as substitutes, being selected to ensure the representativeness of income and respiratory illnesses’ prevalence at the whole perimeter level. The shares of eight combinations of two genders and four age intervals (0 to 4, 5 to 17, 18 to 65, above 65) in the study region’s population were moderately altered to end with considerable numbers of sampled children and elderly. Such percentage quotas, after multiplying by 18, which was the number of interviews a CHA could support, were informed to such professionals, who were instructed to write down a patient’s list with a size two to fourfold that of each absolute quota. Research assistants then drew randomly, for each age–gender group, a number of individuals matching the quota. The interviews were then conducted in interviewees’ houses, and whether selected individuals were not found or refused to participate, the next listed subject of the age–gender group was approached (this was repeated in case of a new failure). Enumerators were accompanied by CHAs in interviews, which both minimized rejection and helped, whenever needed, with rephrasing questions in ways more understandable to respondents.^6^

A total of 519 valid interviews were conducted for the purposes of this paper. In 278 interviews, adults responded about illnesses acquired by them, and in the remaining 241, they responded about illnesses acquired by their children. Caregivers, generally mothers or fathers, were interviewed on behalf of children.

Now turning to the questionnaire, its first module retrieved descriptive data on the most recent illness episode, including (i) a sequence of yes/no/can’t remember questions on whether 21 symptoms were felt,^7^ (ii) a three-point Likert scale on the subjective pain experienced, (iii) the symptoms beginning date and duration, (iv) whether medical care was sought and (v) diagnosed illness.^8^ Children aged above 5 years were asked to confirm caregivers’ responses. The third module asked about age, household size, years of schooling (according to the Brazilian educational system), and household income. The latter was asked in a comprehensive way comprising six main income sources. This was complemented by the inquiry on the ownership of durable goods that were highly correlated with income according to the National Household Survey [57]. Additional questions covered smoking, health insurance, distance to the health unit where treatment for the first module’s illness could be found, and seven serious lung diseases (asthma, bronchitis, lung cancer, chronic obstructive pulmonary disease, emphysema, pneumonia and TB).

### Cough survey

From December 2019 to the middle of March 2020,^9^ a new survey designed as an improvement of the one described in the previous section also was run in Acre state, but this time it also included its second most populous municipality, Cruzeiro do Sul.^10^ The survey consisted of a discrete choice experiment estimating the WTP for a hypothetical remedy^11^ able to reduce the number of days with dry cough. Thus, its main particularity was the focus on one single respiratory symptom, which was selected from medical literature due to its high recurrence across respiratory illnesses [58, 59].^12^ Dry cough was considered because its suppression is more commonly targeted by treatment than wet (with phlegm) cough [62–64], which made the selection of the attributes of the remedy easier (with antitussive drugs being the focus).

The same sampling and children inclusion approaches of the respiratory illness survey were adopted. The modules used in this paper also were near-equivalent to those of the previous survey, except for focussing on the latest dry cough episode and inquiring about income in the form of eight intervals,^13^ whose midpoints were used in the analysis, instead of the open-ended format adopted in the previous survey. Before the final stage of the survey, a prepilot comprising 33 interviews was conducted in July 2019, and a pilot with 54 interviews was conducted in December 2020. The latter is included here as part of the analysed data due to minor differences with the final-stage questionnaire.

### Data quality and analysis

In the respiratory illness survey, two research assistants were trained by the first author and monitored in the field during the pilot wave. These last-year undergraduate students also were qualified to train enumerators. All questionnaires were revised after they were applied, and if needed, the enumerator returned to respondents’ houses, or the questionnaire was discarded. In both cases, the decision was based both on the missing or ill-filled fields and the last questionnaire module, the latter filled out by the enumerator alone, as an assessment of the quality of the responses received. In the cough survey, similar procedures were adopted, with the exception that the whole team was trained by the first author himself (except for one enumerator who conducted 18 interviews).

Standard central tendency, position and dispersion statistics were applied to describe the data. The proportion difference test, based on the standard normal distribution, was applied to compare the adult and child subsamples. Econometric analysis was pursued with standard index multivariate regressions for discrete dependent variables (whether health care was sought), whose disturbances followed either the Gaussian cumulative distribution function (“probit”) or the logistic function (“logit”). Estimators’ standard errors were robust for heteroscedasticity ([65], Chap. 14, [66], Chap. 15). Only models that were globally significant, as informed by a chi-square test of nullity for all covariates’ coefficients ([66], Section 15.5.1), are reported.

## Results

### Surveys’ description

In the respiratory illnesses survey, the average age and income were 30 years and 2.5-fold the minimum wage, respectively, and the frequencies of respondents receiving poverty alleviation cash transfers and with a lower secondary educational level were 30 and 62%, respectively (Table 3). The latest respiratory illnesses reported by participants caused 10 symptoms, lasted 10 days and inflicted moderate to extreme pain in 95% of cases. Only 14% of the episodes occurred in the dry season (i.e., from June to September, in the case of Acre state). Only 38% of respondents sought health care, 32% when the illness was acquired by an adult and 46% when a child was the patient, a statistically significant difference (*p* value < 5%). A specific disease was diagnosed in 79% of cases, and influenza-like illness was the most recurrent diagnosis (64%), followed by pneumonia (12%) and then asthma (6%), with these three collectively accounting for 82% of diagnosed diseases (Table B.1 in the Additional Information document). Viral or bacterial aetiology was dominant (79% of cases). All symptoms with high frequency (> 50%) either attacked the nose or throat or caused cough (dry and wet), fever, headache, body aches, shortness of breath or malaise.

**Table 3:** Statistical summary of variables (respiratory illness survey)

Variable	Whole sample		Only children	
	Mean (SD)	Min-max	Mean (SD)	Min-max
Sought medical care	0.38 (0.49)	0–1	0.46 (0.5)	0–1
Illness’ duration	9.83 (14.07)	1–180	8.21 (6.38)	1–60
Illness’ number of symptoms	10.39 (4.1)	1–20	10.09 (3.87)	2–19
Illness caused extreme pain	0.18 (0.38)	0–1	0.14 (0.34)	0–1
Age	29.61 (23.93)	0.33–89	8.28 (4.87)	0.33–17
Age squared	1448.29 (1828.93)	0.11–7921	92.19 (87.24)	0.11–289
Female	0.5 (0.5)	0–1	0.5 (0.5)	0–1
Ever had lung disease	0.37 (0.48)	0–1	0.35 (0.48)	0–1
Years of schooling	7.83 (4.3)	0–15	8.81 (3.78)	0–15
Household income (log.)	7.49 (0.75)	4.61–10.17	7.46 (0.76)	4.61–10.17
Household size	3.98 (1.85)	1–15	4.51 (1.65)	1–15
Poor	0.29 (0.45)	0–1	0.4 (0.49)	0–1
Has health insurance	0.11 (0.31)	0–1	0.1 (0.31)	0–1
Distance to health unit (metres)	1535.24 (1999.03)	14.94–13,648.26	1660.2 (2084.22)	25.66–10,023.73
Illness occurred in the dry season	0.13 (0.34)	0–1		
Female * household size	2.03 (2.42)	0–13		
Mother answered			0.74 (0.44)	0–1
Father answered			0.11 (0.32)	0–1

Observations amounted to 519 for the whole sample and 241 for children-only

In the cough survey, income was smaller, twice the minimum wage, in line with a higher rate of poverty of 47%,^14^ and age and education were near-equivalent to the data from the respiratory illness survey (Table 4). On average, the most recent dry cough episode lasted 10 days, caused moderate to extreme pain in 78% of cases and occurred in the dry season in 38% of cases. The overall health care seeking rate was 33%: 26% for adults and 39% for children. A significant difference was found at the 10% significance level, and the rate of (pharmacy-purchased) medication was 66%: 57% for adults and 75% for children, with a statistically significant difference at the 5% level. A specific disease was diagnosed in 66% of cases, with influenza-like illness again being the most recurrent diagnosis (67%), followed by pneumonia (8%) and asthma (8%). These three diseases amounted to 83% of diagnosed diseases; two cases of TB were reported (Table B.2 in the Additional Information document). Viral or bacterial aetiology was dominant (83% of cases).

**Table 4:** Statistical summary of variables (cough survey)

Variable	Whole sample		Only children	
	Mean (SD)	Min-max	Mean (SD)	Min-max
Sought medical care	0.33 (0.47)	0–1	0.38 (0.49)	0–1
Cough duration	10.36 (12.95)	1–120	9.22 (8.46)	1–60
Cough caused extreme pain	0.23 (0.42)	0–1	0.25 (0.43)	0–1
Age	27.67 (21.92)	1–81	9.44 (4.66)	1–17
Age squared	1243.24 (1588.51)	1–6561	110.52 (89.25)	1–289
Female	0.52 (0.5)	0–1	0.54 (0.5)	0–1
Ever had lung disease	0.3 (0.46)	0–1	0.28 (0.45)	0–1
Years of schooling	7.57 (4.29)	0–15	7.99 (4.25)	0–15
Household income (log.)	7.2 (0.99)	0–9.85	7.2 (0.84)	5.02–9.11
Household size	4.49 (1.85)	1–11	5.2 (1.92)	2–11
Has time to seek health care	0.76 (0.43)	0–1	0.79 (0.41)	0–1
Poor	0.47 (0.5)	0–1	0.56 (0.5)	0–1
Has health insurance	0.09 (0.29)	0–1	0.08 (0.27)	0–1
Time to health unit	14.38 (14.25)	1–100	15.38 (15.31)	2–100
Waiting time in the health unit	89.27 (84.24)	1–600	92.99 (90.82)	1–600
Resorts to nonprofessional care	0.05 (0.23)	0–1	0.71 (0.46)	
Cough occurred in the dry season	0.34 (0.47)	0–1		
Female * household size	2.48 (2.8)	0–11		
Mother answered			0.71 (0.46)	0–1
Father answered			0.1 (0.3)	0–1

Observations amounted to 168 and, in the case of children-only, 89

In summary, most respondents were of low to medium income, reported a moderately painful influenza-like illness episode over 10 days and were more likely to seek care and purchase medication for children than for adults.

### Econometric analysis

Three models were estimated for each survey. First, there was a baseline model with all factors suggested by the literature review that could be retrieved in the surveys (as in Table 1 above). Second, there was an extended version of the former, including a female gender vs. household size interaction in order to capture that the more members there are in the household, the smaller may be the time female members allocate to domestic tasks and thus the larger the time available to seek health care (this captures intrahousehold effects as in Table 1). Third, there was a child-only model to test whether the kinship tie between child and respondent, supposedly the caregiver, i.e., whether the latter was the mother, father or other relative, influenced health care seeking (which again captures the influence of intrahousehold phenomena).

The results in Tables 5 and 6 show that health care seeking was mainly driven, considering the two surveys, by health condition characteristics, first of all, subjective pain but also severity as measured by the number of symptoms (significant only in the respiratory illness survey) or duration. Factors that were significant only in one survey corresponded to respondents’ health, specifically whether a lung disease was ever experienced (cough survey), and to individual characteristics, specifically age (respiratory illness survey), and, at last, household characteristics, particularly household size (cough survey). In addition, in the cough survey, whether the symptoms occurred in the dry season increased care seeking, as expected, due to the better condition of roads and thus the easier access to facilities and because in the colder dry season, cough could happen together with more or more severe symptoms [67]. There were three factors whose insignificance was less expected. The first was education, revealing that schooling may not capture health knowledge or that the latter was not significantly related to care seeking in the particular context. The second, income, was due to respondents resorting to free care offered by the public system (which also explains why poverty was irrelevant). The last, distance to health unit, could have been caused by most respondents living close to units (75% within 2 km in the illness survey and 94% within 30 minutes in the cough survey), a consequence of the considerable geographical diffusion of primary care facilities and, probably, of CHAs’ priority towards patients located nearer to facilities in routine home visits.

**Table 5:** Multivariate models, respiratory illness survey (dependent variable is whether medical care was sought)

	pro	log	pro_int	log_int	pro_chi	log_chi
Illness’ duration	0.0083	0.0187	0.0082	0.0185	0.0456*	0.0792*
	[0.0067]	[0.0180]	[0.0067]	[0.0181]	[0.0177]	[0.0309]
Illness’ number of symptoms	0.0624***	0.1010***	0.0625***	0.1011***	0.0861***	0.1428**
	[0.0160]	[0.0266]	[0.0160]	[0.0265]	[0.0253]	[0.0435]
Illness caused extreme pain	0.3723*	0.5957*	0.3743*	0.6009*	0.4777	0.8664
	[0.1604]	[0.2696]	[0.1607]	[0.2703]	[0.3026]	[0.5346]
Age	−0.0531***	− 0.0892***	− 0.0530***	− 0.0894***	− 0.0358	− 0.0754
	[0.0099]	[0.0169]	[0.0099]	[0.0171]	[0.0720]	[0.1206]
Age squared	0.0006***	0.0011***	0.0006***	0.0011***	− 0.0017	− 0.0021
	[0.0001]	[0.0002]	[0.0001]	[0.0002]	[0.0039]	[0.0066]
Female	0.18	0.2838	0.0844	0.0517	−0.038	−0.0826
	[0.1208]	[0.2049]	[0.2987]	[0.5447]	[0.1815]	[0.3071]
Ever had lung disease	0.0577	0.109	0.0586	0.1099	0.0719	0.1224
	[0.1275]	[0.2129]	[0.1274]	[0.2127]	[0.1981]	[0.3296]
Years of schooling	−0.0095	−0.0179	− 0.009	− 0.0165	− 0.0332	−0.0595
	[0.0174]	[0.0293]	[0.0175]	[0.0295]	[0.0266]	[0.0460]
Household income	−0.0746	−0.1285	− 0.0758	−0.1319	− 0.0256	−0.0255
	[0.0906]	[0.1505]	[0.0908]	[0.1510]	[0.1331]	[0.2265]
Household size	−0.0629+	− 0.1105	−0.0747	− 0.1427	0.0218	0.0345
	[0.0381]	[0.0680]	[0.0575]	[0.1141]	[0.0560]	[0.0951]
Poor	0.1061	0.1763	0.1076	0.1806	0.1717	0.2984
	[0.1375]	[0.2255]	[0.1375]	[0.2253]	[0.2000]	[0.3427]
Has health insurance	0.1582	0.3125	0.1596	0.3169	0.0919	0.131
	[0.1990]	[0.3313]	[0.1993]	[0.3322]	[0.3014]	[0.5054]
Distance to health unit	0	0	0	0	0	−0.0001
	[0.0000]	[0.0001]	[0.0000]	[0.0001]	[0.0000]	[0.0001]
Illness occurred in the dry season	−0.0629	− 0.0982	− 0.0671	−0.1078		
	[0.1746]	[0.2927]	[0.1749]	[0.2942]		
Female * household size			0.0241	0.059		
			[0.0700]	[0.1302]		
Mother answered					−0.08	−0.0907
					[0.2449]	[0.4057]
Father answered					−0.2554	− 0.339
					[0.3469]	[0.5909]
Intercept	0.2277	0.4722	0.2765	0.6082	−0.5058	−0.8943
	[0.6733]	[1.1343]	[0.6981]	[1.1981]	[1.0677]	[1.8154]
N	519	519	519	519	241	241
r2_p	0.1155	0.117	0.1157	0.1174	0.1653	0.1669
chi2	66.1068	59.975	66.0322	59.6461	40.7541	36.3801
p	0	0	0	0	0.0003	0.0016
ll	− 305.6186	−305.0764	−305.5477	− 304.936	− 138.804	−138.5359

Standard errors in brackets; “pro” denotes probit and “log” logit models, the first two columns contain the baseline model, the model with female * household size interaction is indicated with “int” and the children-only model with “chi”. Pseudo-R^2^ is denoted with “r2_p”, the statistic and *p* value of the chi-square global significance test as “chi2” and “p”, and the log-likelihood as “ll”

**Table 6:** Multivariate models, cough survey (dependent variable is whether medical care was sought)

	pro	log	pro_int	log_int	pro_chi
Cough duration	0.0211*	0.0345+	0.0202*	0.0328	0.0547**
	[0.0096]	[0.0191]	[0.0100]	[0.0204]	[0.0202]
Cough caused extreme pain	0.8242**	1.3778**	0.8748**	1.4805**	0.6636+
	[0.2855]	[0.4963]	[0.3012]	[0.5317]	[0.4021]
Age	−0.0459+	−0.0739+	− 0.0458*	− 0.0750+	− 0.3453*
	[0.0234]	[0.0432]	[0.0228]	[0.0410]	[0.1735]
Age squared	0.0006+	0.0009	0.0006+	0.0009	0.0166+
	[0.0003]	[0.0007]	[0.0003]	[0.0006]	[0.0090]
Female	−0.0791	−0.148	−0.743	−1.3225	0.187
	[0.2346]	[0.4182]	[0.7103]	[1.2908]	[0.3326]
Ever had lung disease	0.6274*	1.0551*	0.5731*	0.9722*	0.4161
	[0.2627]	[0.4666]	[0.2662]	[0.4708]	[0.3831]
Years of schooling	−0.0409	− 0.0674	− 0.0435	− 0.0708	− 0.0186
	[0.0323]	[0.0555]	[0.0319]	[0.0540]	[0.0379]
Household income	−0.0096	−0.0428	− 0.0162	−0.0506	− 0.3177
	[0.1333]	[0.2424]	[0.1314]	[0.2346]	[0.2529]
Household size	0.2210**	0.3729**	0.1237	0.2002	0.1513+
	[0.0769]	[0.1378]	[0.1259]	[0.2264]	[0.0874]
Has time to seek health care	0.0565	0.0548	0.0388	0.0259	−0.1728
	[0.2674]	[0.4498]	[0.2665]	[0.4471]	[0.3553]
Poor	0.0394	0.05	0.0233	0.0109	−0.0046
	[0.2919]	[0.5255]	[0.2930]	[0.5267]	[0.3930]
Has health insurance	0.4803	0.7895	0.4547	0.7318	−0.0821
	[0.4039]	[0.6859]	[0.4011]	[0.6775]	[0.6626]
Time to health unit	−0.0006	0.0004	0.0019	0.0044	−0.0035
	[0.0098]	[0.0198]	[0.0099]	[0.0195]	[0.0107]
Waiting time in the health unit	−0.002	− 0.0034	− 0.0022	− 0.0037	−0.0006
	[0.0015]	[0.0026]	[0.0015]	[0.0027]	[0.0016]
Resorts to nonprofessional care	−1.0307+	− 1.7512+	− 1.0017	−1.7174	
	[0.6113]	[1.0464]	[0.6240]	[1.0696]	
Cough occurred in the dry season	0.7812**	1.2846**	0.8089***	1.3378**	
	[0.2439]	[0.4284]	[0.2455]	[0.4315]	
Female * household size			0.1463	0.2572	
			[0.1494]	[0.2683]	
Intercept	−1.3504	− 2.0615	−0.8489	− 1.1943	2.0861
	[0.9920]	[1.7160]	[1.0788]	[1.8517]	[2.1098]
N	168	168	168	168	89
r2_p	0.2538	0.249	0.2585	0.2539	0.208
chi2	55.8248	46.4543	55.6801	46.5984	24.4023
p	0	0.0001	0	0.0001	0.0409
ll	−79.2711	−79.7813	−78.7682	− 79.2529	−46.8759

The children model with dummies indicating the child’s relative who responded to the questions was not globally significant either in logit or probit form and thus was omitted. Without such variables, only the probit model was globally significant

Among the remaining nonsignificant factors, waiting time inside the health unit (cough survey) was irrelevant, probably due to insufficient variation (58% waited up to 1 h). Resort to nonprofessional health care (cough survey), which mostly captured household-made medication, was nonsignificant, probably because it was not a question posed to all respondents but, instead, a spontaneous statement made as part of the choice experiment whenever medication was not chosen. This probably biased the variable’s significance downwards. Household-level phenomena, captured by the interaction of female gender and household size and by the relative who answered on behalf of the child (supposedly the caregiver), did not significantly influence the health care-seeking decision.

## Conclusions

### Connections of findings with the revised literature

In this section, the main results are concisely linked with the literature classified in Table 1 for each class of factors influencing health care seeking. The macroeconomic factors class is omitted for not being at a scale compatible with a survey on individuals, but they are addressed in the next subsection (on other factors and policy).

#### Individual characteristics

The higher propensity to seek care and purchase medication when illnesses or cough were experienced by children is novel evidence in health care-seeking research, as it was found in only two papers, which, nevertheless, did not highlight the evidence’s relevance (see [4, 26]). The practice of relying on surveys targeted either to children or adults, but not to both, was dominant in the remaining papers consulted. Notwithstanding, and despite biases from different survey goals and methods, the average was computed across papers reporting health care seeking rates, revealing a three percentage point larger number for children (79% vs. 76%). Our finding thus is not only innovative but also valid beyond the region of study. Nevertheless, as [22] emphasize, the higher rate of demand for medical services for children should not be presumed to translate into a higher rate of cure, as the latter depends on the appropriateness of the treatment received, which could differ between children and adults. Regardless, it may be claimed that there is a rationale for children- and adult-specific measures to be taken as part of a policy to expand health care demand, which is further developed in the next subsection.

#### Health level and condition

The significance of duration, severity^15^ and subjective pain and the insignificance of individual, household and facility characteristics in models explaining whether an episode of respiratory illness or dry cough motivated a medical visit reveal that subjective, rather than objective, factors play a key role in driving health care seeking. This is in line with the revised literature. In [7], the expectation that relatives and friends would support care seeking was a significant predictor of opting for it. In addition, symptoms that were believed to be of a somatic rather than psychological nature and thus less stigmatized were more likely to trigger a stated intention to seek health care. Similarly, disease stigma was emphasized as a demand-side delayer of TB treatment by [5, 25]. These two papers imply that subjective misevaluation of disease severity, which may be related to the level of pain experienced, affects care seeking. In [29], the subjective values of administration mode, dosing, risk of adverse events and the interruption of daily routine were shown to influence demand for medication. Objective factors such as gender, income and access to health facilities also were found by other studies to be insignificant predictors [5, 6, 22, 29].

#### Health knowledge

Notwithstanding the insignificance of objective factors not being a peculiarity of this paper, education is generally found to be relevant [6, 29], an exception being [22]. Education, nevertheless, is not 100% correlated with health literacy^16^ (i.e., the capacity to recognize symptoms and illnesses and to make decisions to prevent or manage them), with the latter a stronger force behind health care demand [7]. Therefore, the findings obtained should not be understood as an irrelevance of health knowledge as a predictor of health care demand. In fact, the main implication is the need for the set of detailed questions measuring health literacy proposed by [68] to be incorporated into a future survey.

#### Intrahousehold relationships, gender and communities

Household-level behavioural variables were insignificant, except for household size in the cough survey, a perhaps too narrow measure of intrahousehold interactions, which also was found to be relevant by [6, 26]. Nevertheless, the insignificance of the more precise measures, namely, caregiver and respondents’ gender, and of the interaction of patient’s gender and household size defies [24] qualitative evidence that gender-biased children caregiving and household labour division affect whether and how fast a health professional was sought. It also challenges the finding of widowed and divorced TB patients being less likely to seek care [6]. The reason for the discordance seems to lie in the different measures of household processes employed here and in the cited papers, since different mechanisms are captured in each case. Female domestic labour burden and kinship ties between children and caregivers were prioritized in this paper, whereas a wider set of interaction situations were investigated in [6, 24] looked strictly to marital status. The more comprehensive assessment of [24] and the household care-seeking decision theory behind it suggests that such mechanisms are not necessarily contradictory, so that a final answer on the influence on household-level interactions is left for a survey designed to isolate the effects of specific mechanisms.

#### Access to care

The insignificance of distance to health unit is at odds with [26] but not with [22, 25]. Notwithstanding, the consequence of the evidence observed here, that travel cost does not disable access to health services, seems to be restricted not only to countries with nationwide coverage of the public health system (such as Malawi [26];) or of a specific health programme (such as the Ethiopian TB programme [5];) but also to urban regions within such countries. Indeed, one limitation of the surveys analysed here is their circumscription to urban centres, whereas in rural areas, health units are scarcer.^17^ This is in accordance with the opposition between the null and the negative influences of distance to facilities found, respectively, in this paper and in [26], which is probably due to the latter capturing a mostly rural population. That this explanation does not apply to the null influence found in the strictly rural survey by [22] is clarified by the authors, who argue that the straight line distance they relied on was not appropriate to measure travel cost in rural areas, as it ignored geographical obstacles. In summary, the lesson from [26], that travel cost is a financial barrier whose impact on limiting demand for health facilities may be relevant even without user charges applied, should not be deemed invalid, especially at the national level.

#### Nonprofessional care

The lack of influence of the resort to nonprofessional care, in the form of home-made medication, contradicted the results by [6, 24, 26]. According to the authors, reliance on traditional healing either delayed (in the case of TB patients) or completely prevented treatment (for infant diarrhoea, malaria and pneumonia). One explanation for the divergence is that traditional and professional treatment are perhaps more likely to complement rather than substitute each other in the case of the mainly viral acute respiratory illnesses in this paper’s surveys rather than for the chronic bacterial diseases reported in [6, 22, 24], with the latter paper also having investigated malaria and diarrhoea. Another explanation is that the option for relying exclusively on traditional medicine was due to other forces whose intensities were lower in the Acre state. Two examples are affordability of medical services [24], which is not a barrier, as services are freely supplied, and travel cost [22, 24], which had no influence in this paper’s region of study.

### Other factors not controlled for and policy implications

Some words are merited on the macroeconomic factors that were not directly controlled for. The linking of results with the macroeconomic evidence provided by [8, 27] suggests that subjective factors preventing respiratory ill individuals from seeking health care (such as pain being sensed as tolerable) have macroeconomic implications. The duration of the temporary human capital reduction is extended at the individual level and, possibly, also at the social level in the case of viral and bacterial diseases [5]. Economic growth may be diminished through the long-run relationship that connects it with health care demand and health level, according to the mentioned articles.

Any consideration of implications for policy-making should be made in light of two key pieces of evidence. First, the low level of care seeking, with at most 40% of respondents seeking care, in contradiction with the severity of the respiratory conditions respondents experienced, which lasted 10 days, involving ten symptoms on average, or at least dry cough, and mainly causing moderate or extreme pain. What should be noted is not at odds with previous studies ([3, 4, 7]).^18^

The second relevant information is the most frequent classes of diseases in the target population. In this sense, even with the disease most frequently reported by participants (influenza-like illness) belonging to the acute viral category, thus requiring mild medical intervention, the second most reported was pneumonia, a contagious disease for which treatment is needed, thus avoiding complications that could lead to hospitalization and even death [50], with the latter especially likely in the case of small children [70]. Contagion inside the household, nursing homes and local communities also is a worry [50]. A considerable frequency of diagnosed pneumonia (7%) also was observed in Acre state’s ambulatory records (Table 2),^19^ together with a 30% frequency of acute pharyngitis and tonsillitis, which also are transmissible diseases.

Therefore, synthesizing the two pieces of evidence, the health care-seeking gap is both sizeable and a cause of avoidable human and economic costs. Pneumonia transmission is a clear demonstration of such costs, but influenza-like illnesses, pharyngitis and tonsillitis, even requiring milder medical intervention, may lead to work absenteeism, lower productivity and failure to undertake household tasks, which compress human capital and GDP [71]. Whether it pays off to foster increased health care seeking for these nonsevere diseases depends on the intensity of the trade-off between the cost imposed on the health system and on households and the remaining economy. Even being out of the scope of this paper to measure such intensity, by highlighting the need for assessing it as part of the process of improving Acre state health policy, an already useful policy contribution is made.

The results also support the conjecture that supply-side policy recommendations, such as performance-based increases in government expenditures on health care (suggested by [27]), may bring less-than-expected results due to demand-side barriers. One clear barrier is the subjective underestimation of respiratory illnesses’ consequences, both for own health and for the broader society, as suggested both by a rate of care seeking not above 40% and by the dominant role of subjective predictors. In this regard, a two-pronged policy strategy may be successful. First, regarding respiratory illnesses that are both severe and transmissible, implementation of microeconomic behavioural policies seeking to change subjective perceptions of symptoms and illnesses, via nudges [23] but also through educational health interventions (as proposed by [6, 7]), is advisable. One possibility, suggested by the lower likelihood of adults seeking care compared to children, is CHAs’ educational actions stressing that adults’ illnesses, if contagious, could be acquired by their children. Influenza vaccination campaigns that achieve over 80% coverage in Acre state [31] also could better inform the population about the symptoms and contagiousness (as recommended for TB by [6]) of severe diseases. A specific study on alternatives to increase health care usage during the wet season is needed, which requires assessing the possibility of temporarily expanding health workers’ teams in rural areas when rain is intense enough to prevent rural-urban displacements.

Regarding nonsevere diseases, such as influenza-like illnesses, campaigns informing about household-based management would probably be more cost-effective, avoiding the overload of an already deficient health system (according to Section 2.3). In particular, primary care home visits could be intensified by hiring more CHAs. However, again, assessing the trade-off between the health system and economic costs is needed to know whether some of the recommendations in the previous paragraph also are reasonable for nonsevere diseases.

Two key limitations are worth stressing as gaps to be filled by future research. First is the circumscription to urban centres, which, albeit concentrating most of the population, may have diminished accuracy in the estimation of the significance of potential predictors, with travel cost being a clear example. Relatedly, Brazilian Amazon-wide valid estimates of predictors’ influences would require expanding the survey to the other eight states integrating the region. Second, health knowledge and reliance on traditional medicine were proven to require specific and detailed sets of questions, the same also being true for intrahousehold interactions and decision-making. Finally, the results achieved here are generalizable only to other developing countries that also have a public health system targeting universal coverage and offering free health care (as Malawi [26]).

## Supplementary Information

**Additional file 1.**

## Data Availability

The datasets generated and/or analysed during the current study are not publicly available due the confidentiality commitment contained in the informed consent document signed by participants, which forbids public data sharing, but are available from the corresponding author on reasonable request.

## Abbreviations

TB: Tuberculosis
CHA: Community health agent
WTP: Willingness to pay
SD: Standard deviation

## Footnotes

1 This estimate considers, from the nine Amazonian states, only the six belonging to the Brazilian Northern region.

2 Idem.

3 As stated in group interviews with CHAs conducted in Rio Branco and Cruzeiro do Sul in August and September 2019.

4 WTP was estimated as part of a broader research project about policy alternatives to reduce atmospheric pollution from agricultural fires (as detailed in [56]).

5 The help of three CHAs was sought in each neighbourhood, a number that was reduced in the few cases of small neighbourhoods.

6 In addition, CHAs’ presence ensured the safety of the undergraduate enumerators, since criminal activity was frequent in some localities according to CHAs and primary care staff.

7 For the reader interested in knowing which symptoms were inquired, the most recurrent among them are reported in Region of study below.

8 Other questions not used in this paper, for being out-of-scope or for being responded to by an insufficient number of participants, were Likert scales of agreement with four potential causes of the illness and, only in the case in which care was sought, the time waited in the health facility and missed days of work.

9 It is clarified that the cough survey was completed before confirmation of the first COVID-19 case in Acre state (the first case was detected on 19 March and the last interview occurred on 18 March). It was exactly because COVID-19 was starting to spread in southeastern Brazil (where the first author, who was legally responsible for the survey before the Brazilian Ethical Council, was based) that the survey was stopped, avoiding the exposure of enumerators (undergraduate students) and community health agents to risk.

10 Due to the COVID-19 pandemic, interviews were interrupted, resulting in a sample inferior to the planned level but still sufficient for econometric analysis.

11 Medication should lead to a more realistic and conservative estimate of WTP to avoid a respiratory illness than the prevention yielded by vaccination.

12 It also is related, in the literature, with the inhalation of fine particulate matter released by the combustion of biomass in general, and, in particular, by agricultural burnings ([60, 61]).

13 The intervals were: (0) zero income, (1) zero to half minimum wage (MW), (2) half MW to 1 MW, (3) 1 to 2 MWs, (4) 2 to 3 MWs, (5) 3 to 4 MWs, (6) 4 to 5 MWs, (7) 5 to 10 MWs, (8) > 10 MWs.

14 Lower income and higher poverty in the cough survey were due to the inclusion of the Cruzeiro do Sul municipality, where 54% of interviews occurred.

15 Duration and number of symptoms may be objectively measured but are here considered subjective for being directly related with the discomfort experienced.

16 In the health literacy scale used by Suka et al. (2016 and 2013), besides capacity to read and understand information, which are skills directly related with education, there also are less related components, such as proneness to seek information about disease and treatments and whether the credibility of obtained information is checked or not.

17 With the average per capita number of ambulatory plus hospital beds being 37% larger in Amazonian municipalities above 100,000 inhabitants by November 2021 [10, 15].

18 According to [59], “many patients do not seek medical attention” for cough, and [69], which claimed that “probably the majority of patients with cough, certainly acute cough, go to a pharmacy rather than a physician and therefore escape epidemiological surveys”.

19 It is helpful to describe the main differences between the surveys and the ambulatory records. The geographical reach of the former is broader, as it captures the whole state, including rural areas. It also tends to have more precise diagnosis information, as it is not based on patients’ memory, as it is in the case of the surveys, but rather on a compilation of physicians’ reports. Nevertheless, in the surveys, inhabitants were visited in their houses by interviewers, whereas it was their choice to visit a health unit and thus to be captured in the ambulatory dataset.
