# Supplementary material for: Drivers of respiratory health care demand in Acre state, Brazilian Amazon: a cross-sectional study
Source: BMC Public Health. 2022 Sep 24;22:1821. doi: 10.1186/s12889-022-14171-z (PMC9509621; doi:10.1186/s12889-022-14171-z)
Supplement: Supplementary file 1 — Additional file 1. [file 12889_2022_14171_MOESM1_ESM.docx]

**Additional information for “Drivers of health care demand for respiratory illnesses in Acre state, Brazilian Amazon: a cross-sectional study”**

# A Summary of references

Here, detailed summaries of the papers basing the taxonomy found in table 1 of the main text are provided.

1. In [1] it is asked which of four stages of the process of seeking care from a physician or similar professional were more likely to be reached by rural Nigerian households and which factors influenced decisions in each stage. Parents, together with community leaders and health care providers, were surveyed about the stages, being them: (1) recognition of illness by the parent, (2) household choice of action, (3) use of care provided by community and (4) use of care provided by a chemist or a health facility. This framework is enlightening for revealing that health care demand results from multiple individual and collective decisions, made within different organizational arrangements (household and community) and involving professional and non-professional providers. Results showed that parents were able to correctly identify illnesses from symptoms and also to point to potential causes, except for pneumonia which was wrongly linked to weather and food intake. Intra-household labour division between genders was relevant, with the father engaged in raising funds and the mother providing physical care and taking children for treatment. Mothers were found to have a leading role in initiating the care seeking process and in moving through the stages and prompt communication between parents increased likelihood of care seeking. Self-made medication from herbs, but also resort to a chemist (a non-professional pharmaceutical), were motivated not only by community traditions but by limited affordability. But when such alternatives yielded non-satisfactory results, health facilities were visited, what was also more likely for Christians.
2. In [2], it is showed that stigma of tuberculosis (TB) was highly recurrent among those affected by the disease and to be statistically related with female gender (+^[[1]](#footnote-1)^), subjective severity of disease (+), anxiety (‑), social support (‑) and good doctor-patient communication (‑). Stigma, which attests a wrong understanding of the disease (whose contagiousness is overestimated) and manifests as a fear of revealing own health condition (and many lead to isolation), reduces the likelihood to seek medical treatment. In fact, stigma is an extreme case of subjective misevaluation of disease severity by patients, with other papers such as [3], having detected underestimation of severity (such as interpreting symptoms as due to common cold) to also delay care seeking.
3. [3] disentangled demand (patient) and supply (health system) sources of delay in TB treatment in Ethiopia, estimating the former to be the main source, contributing with 21 days of delay due to late care seeking. The latter source contributed with 12 days due to diagnosis and treatment initiation delays. Demand side drivers of delay were unaffordability of treatment and even poverty, reliance on non-professional care (traditional healers and chemists) and unawareness (including “the expectation that symptoms will resolve by themselves”). Supply side causes were scarcity of human (health professionals) and physical resources (laboratories) and failures of community health workers to identify TB cases and properly refer patients to physicians. Women were more subjected to delay due to high stigma, discrimination and overburden of domestic work. The paper highlights the importance of well-trained community health workers in detection of TB cases and of interventions to reduce treatment cost and increase awareness.
4. Similarly to the previous paper, [4] also investigated predictors of delay in seeking TB treatment in Ethiopia, with a quantitative survey with 382 patients from public health facilities. The study adds to [3] in finding that rural residency (+), self-medication (+), elderly age (+), a walking distance of more than 30 minutes to facility (+), family larger than three members (+) and windowed and divorced marital statuses (‑) were significant predictors of demand-side delay. Delay on diagnosis was larger whether a general practitioner was initially visited and whether chest X-ray was not used, showing again the importance of human and physical resources. Gender was not a relevant predictor of demand-side delay, in contradiction with previous studies and also with [3], what is explained on the basis of growing women empowerment in Ethiopia (in line with [5]). That delay increased with family size was explained by overburden of “family responsibilities” and exposure to severer “socio-economic hardship” preventing care seeking. Authors also argue in the discussion that recognition of TB symptoms is a relevant factor in care seeking and that reliance on self-medication and traditional medicine delayed treatment – it is stated that half of developing countries’ population rely first on “traditional healing systems” ([4], p.10).
5. In [5] it is found that, in rural Malawi, healthcare demand, measured both as healthcare seeking and out-of-pocket expenditure, was significantly predicted by distance to health facility (‑), age (‑), chronic disease (+), household size above five members (‑), female gender (+), illness duration (+), limitation in capacity to pursue routine activities (‑), wealth (+) and urban residency (+). This was based in a 1,639 sample household survey. The self-selection bias inherent to the observability of expenditure only for those seeking care was addressed with Heckman's technique. Women being more likely to seek care opposed other studies (including [2], [3] and [4]), what suggests that the gender’s lack of autonomy to pursue the action may be less limited in particular contexts. A second household process detected was priority in healthcare seeking attached to productive age individuals especially in allocation of funds for out-of-pocket expenditures. The paper highlights that universal coverage of health care in Malawi was not yet achieved even with free provision, due to (staff and drug) bottlenecks in the system perpetuating out-of-pocket disbursements, but also due to indirect costs such as that of traveling to health facilities, which proved substantial in the survey and larger in average than out-of-pocket expenditure.
6. In [6] it was investigated the drivers of psychological healthcare seeking in Japan with a “stated action” survey in which patients, being subjected to treatments in the form of four symptoms (two psychological and two somatic symptoms, all of them associated with depression), responded by choosing one amongst ten pre-defined actions ranging from seeking advice from relatives or in the internet to visiting a physician, with the option of doing nothing also included. The strongest predictor was judging that relatives and friends would agree with care seeking. Other factors increasing significantly care seeking were mental health literacy or the ability to obtain and use information in order to ensure good health, female gender, having visited a mental health professional and interacting with mentally ill people, benefiting from social capital in the form of good communication with neighbours and non-stigmatization of disease ‑ the latter was only significant in the particular case of seeking formal care. Authors argued that symptoms that could be interpreted as due to somatic, rather than mental causes ‑ which are more prone to be stigmatized ‑, were more likely to result in demand for health care. Interventions to expand mental health literacy, especially inside households, are recommended.
7. [7] ran a discrete choice experiment (DCE) in the UK in which choices among different modes of drug administration and their attributes demonstrated that basing development of medication in patients' preferences could bring efficiency gains not only to developers but to healthcare providers. The attributes of DCE alternatives included, besides administration route (intravenous, subcutaneous, intramuscular and needle-free), dosing and monetary and non-monetary costs. Results suggested that demand for medication, and for related healthcare, could be increased by less invasive administration modes than intramuscular that could be pursued by patients themselves at lower dosages and imposing lower risk of adverse events and disruption of daily routine. Medication price was significant but small. Both welfare of patients would be increased and expenditure by health care system would be reduced by introducing a drug more compatible with the preferences measured. Therefore, the paper demonstrated the relevance of demand-side evaluations of health care acquisition in revealing improvements in health services and related policy.
8. [8] evidenced conditional convergence on per-capita health expenditure within subgroups of OECD countries from 1975 to 2004, and thus the role of income growth in boosting aggregate health demand. Of three potential drivers of the latter, only technological progress was empirically significant, due to life/health-enhancing new medical products (and services) being normal goods and also because the larger the income the more rewardable is investing in increased future health and working capacity. The other two factors that proved empirically irrelevant were ageing and health insurance. Health expenditure was also found to have less-than-unitary elasticity with per capita GDP, attesting that healthcare is a necessary good.
9. In similar fashion, [9] applied, in the macroeconomic scale, Granger-causality tests to GDP and governmental health care expenditure, concluding on one directional causality running from the former to the latter in Subsaharan Africa and bidirectional causality in central Africa. The two variables were thus connected across time by a long run relationship capturing both a human capital enhancing mechanism through which GDP increased health care expenditure and a public budget mechanism in the opposite direction. The implication of the study for the purposes sought in this paper is demonstrating that increased healthcare demand has macroeconomic consequences (via the enhanced human capital channel). The authors argue for supply-side policy in increasing governmental health care expenditure with performance-based financing.
10. [10] estimated macro-level healthcare demand of 36 Asian countries showing that the service was a necessary good (as in [8]), attesting both the role of income in determining demand and the propensity of population to respond to a fall in income with a less than proportional reduction in quantity demanded. A long-run (cointegration) relationship was found to exist among healthcare demand, income and health level as proxied by infant mortality, with the latter negatively impacted by increases in the other two variables.
11. [11] estimated, with a Hong-Kong survey, rates of care seeking for specific respiratory and gastrointestinal symptoms evidencing rates of 78%, 60% and 50% for fever, rash and shortness of breath, as compared with an overall 40% rate across all symptoms which included milder ones such as runny nose, sore throat, fatigue, etc.). Which may be interpreted as a trend of care seeking increasing with symptom severity, in line with the finding that western medicine, which, according with the authors is understood as more “powerful and quick”, was more likely to be relied on in the case of acute symptoms than in case of “gradually developing” chronic symptoms. Those immediately seeking care were around 15% and about 40% delayed at least 2 days ([11]). Working-age patients were less likely to seek care.
12. [12] conducted a survey seeking to assess the demand for health care for children with diarrhoea, malaria and pneumonia in four poor rural districts in Sierra Leone. The main factors driving resistance to seek care were traditional medicine and barriers to facility care related with affordability and accessibility. Children with diarrhoea and multiple symptoms were more likely to be taken to a physician, another evidence that care seeking is more probable the severer the symptoms. Also, patients with pneumonia were more likely to seek treatment. Provision of non-recommended treatment was also considerable, both by non-professional and professional health care sources.

# B Classifications of diseases reported in the surveys

**B.1 Classification of diseases reported in the respiratory illness survey**

| **Disease class** | **Approximate aetiology** | **Count** | **%** |
| --- | --- | --- | --- |
| Asthma | Viral, bacterial, allergen-driven, pollution-exposure-driven, but also genetic | 9 | 16,1% |
| Acute bronchitis and bronchiolitis | Mainly viral, but bronchitis may be also bacterial | 2 | 3,6% |
| Bronchitis, emphysema and other chronic obstructive lung diseases | Smoking and air pollution | 8 | 14,3% |
| Chronic diseases of tonsils and adenoids | Bacterial | 0 | 0,0% |
| Acute pharyngitis and tonsillits | Bacterial or viral | 4 | 7,1% |
| Influenza-like illnesses | Viral | 0 | 0,0% |
| Acute laryngitis and tracheitis | Bacterial or viral | 0 | 0,0% |
| Other diseases of the respiratory system | Indeterminate, as multiple diseases are included | 4 | 7,1% |
| Other diseases of the nose and the paranasal sinuses | Idem | 0 | 0,0% |
| Other diseases of the upper respiratory tract | Idem | 0 | 0,0% |
| Other acute infections of the upper airways | Idem | 10 | 17,9% |
| Pneumonia | Bacterial or viral | 19 | 33,9% |
| Chronic sinusitis | Bacterial | 0 | 0,0% |
| Unknown | Does not apply | 33 |  |
| NA | Does not apply | 9 |  |
| Total | | 98 |  |
| Total excluding unknown and NA cases | | 56 | 100,0% |

Notes: in the first column, “Unknown” indicates statements mentioning symptoms instead of a diagnosed disease and the “NA” refers to cases in which a diagnosis was not provided by the physician (in one case, the attempt to see a physician failed).

**B.2 Classification of diseases reported in the cough survey**

| **Disease class** | **Approximate aetiology** | **Count** | **%** |
| --- | --- | --- | --- |
| Asthma | Viral, bacterial, allergen-driven, pollution-exposure-driven, but also genetic | 3 | 8,3% |
| Acute bronchitis and bronchiolitis | Mainly viral, but bronchitis may be also bacterial | 0 | 0,0% |
| Bronchitis, emphysema and other chronic obstructive lung diseases | Smoking and air pollution | 1 | 2,8% |
| Chronic diseases of tonsils and adenoids | Bacterial | 0 | 0,0% |
| Acute pharyngitis and tonsillits | Bacterial or viral | 1 | 2,8% |
| Influenza-like illnesses | Viral | 24 | 66,7% |
| Acute laryngitis and tracheitis | Bacterial or viral | 0 | 0,0% |
| Other diseases of the respiratory system | Indeterminate, as multiple diseases are included | 0 | 0,0% |
| Other diseases of the nose and the paranasal sinuses | Idem | 0 | 0,0% |
| Other diseases of the upper respiratory tract | Idem | 0 | 0,0% |
| Other acute infections of the upper airways | Idem | 2 | 5,6% |
| Pneumonia | Bacterial or viral | 3 | 8,3% |
| Chronic sinusitis | Bacterial | 0 | 0,0% |
| Tuberculosis | Bacterial | 2 | 5,6% |
| Not a respiratory illness | Does not apply | 1 | 2,8% |
| Unknown | Does not apply | 7 |  |
| NA | Does not apply | 11 |  |
| Total | | 55 |  |
| Total excluding unknown and NA cases | | 36 | 102,8% |

Note: the same clarifications in the notes to the previous table apply.

# References

1. Dougherty, L., Gilroy, K., Olayemi, A., Ogesanmola, O., Ogaga, F., Nweze, C., Banerjee, J., Oduenyi, C., & Pacqué, M. Understanding factors influencing care seeking for sick children in Ebonyi and Kogi States, Nigeria. BMC Public Health. 2020; 20, 746. <https://doi.org/10.1186/s12889-020-08536-5>.
2. Chen, X., Du, L., Wu, R., Xu. J., Ji, H., Zhang, Y., Zhu, X., & Zhou, L. Tuberculosis-related stigma and its determinants in Dalian, Northeast China: a cross-sectional study. BMC Public Health. 2021; 21, 6. <https://doi.org/10.1186/s12889-020-10055-2>.
3. Datiko, D.G., Jerene, D., & Suarez, P. Patient and health system delay among TB patients in Ethiopia: Nationwide mixed method cross-sectional study. BMC Public Health. 2020; 20, 1126 <https://doi.org/10.1186/s12889-020-08967-0>.
4. Seid, A., & Metaferia, Y. Factors associated with treatment delay among newly diagnosed tuberculosis patients in Dessie city and surroundings, Northern Central Ethiopia: a cross-sectional study. BMC Public Health. 2018; 18(1), 1-13. <https://doi.org/10.1186/s12889-018-5823-9>.
5. Nakovics, M. I., Brenner, S., Bongololo, G., Chinkhumba, J., Kalmus, O., Leppert, G., & De Allegri, M. Determinants of healthcare seeking and out-of-pocket expenditures in a “free” healthcare system: evidence from rural Malawi. Health economics review. 2020; 10, 1-12. <https://doi.org/10.1186/s13561-020-00271-2>.
6. Suka, M., Yamauchi, T., & Sugimori, H. Help-seeking intentions for early signs of mental illness and their associated factors: comparison across four kinds of health problems. BMC Public Health. 2016; 16(1), 1-13. <https://doi.org/10.1186/s12889-016-2998-9>.
7. Tetteh, E. K., Morris, S., & Titcheneker-Hooker, N. Discrete-choice modelling of patient preferences for modes of drug administration. Health economics review. 2017; 7(1), 1-14. <https://doi.org/10.1186/s13561-017-0162-6>
8. Nghiem, S. H., & Connelly, L. B. Convergence and determinants of health expenditures in OECD countries. Health economics review. 2017; 7(1), 1-11. <https://doi.org/10.1186/s13561-017-0164-4>.
9. Piabuo, S. M., & Tieguhong, J. C. Health expenditure and economic growth-a review of the literature and an analysis between the economic community for central African states (CEMAC) and selected African countries. Health economics review. 2017; 7(1), 1-13. <https://doi.org/10.1186/s13561-017-0159-1>.
10. Abdullah, S. M., Siddiqua, S., & Huque, R. Is health care a necessary or luxury product for Asian countries? An answer using panel approach. Health economics review. 2017; 7(1), 1-12. <https://doi.org/10.1186/s13561-017-0144-8>.
11. Zhang, Q., Feng, S., Wong, I. O., Ip, D. K., Cowling, B. J., & Lau, E. H. A population-based study on healthcare-seeking behaviour of persons with symptoms of respiratory and gastrointestinal-related infections in Hong Kong. BMC public health. 2020; 20, 1-10. <https://doi.org/10.1186/s12889-020-08555-2>.
12. Diaz, T., George, A.S., Rao, S.R., Bangura, P.S., Baimba, J.B., McMahon, S.A., & Kabano, K. Healthcare seeking for diarrhoea, malaria and pneumonia among children in four poor rural districts in Sierra Leone in the context of free health care: results of a cross-sectional survey. BMC Public Health. 2013; 13, 157. <https://doi.org/10.1186/1471-2458-13-157>.

1. Hereafter, when convenient, the direction of predictors' influence is indicated with “‑” and “+” signs, as in table 1 of the main text. [↑](#footnote-ref-1)
